# Quality improvement strategies in the management of poisoning: a systematic review

**DOI:** 10.1186/s12913-026-15038-4

**Published:** 2026-07-18

**Authors:** Veekshitha S. Shetty, Prathvika S. Shetty, Rajalakshmi Rajendran, Shilpa Kadangal, Marva Abdul Khader, Girish Thunga

**Affiliations:** Department of Pharmacy Practice, Manipal College of Pharmaceutical Sciences, Manipal Academy of Higher Education, Manipal, Karnataka 576104 India

**Keywords:** Antidote, Emergency care, Quality improvement, Poisoning, Toxicological stewardship

## Abstract

**Background:**

Poisoning remains a major global public health concern, contributing significantly to morbidity and mortality. The management of poisoning cases is challenged by the absence of standardized treatment protocols, limited diagnostic frameworks, inconsistent antidote availability, and variability in clinical practice. Evaluating quality improvement (QI) approaches is essential to assess current standards of care and identify strategies to optimize poisoning management. This systematic review aimed to evaluate quality management in patients with poisoning by assessing the impact of quality improvement strategies on clinical processes and patient outcomes.

**Methods:**

This review was conducted in accordance with the Preferred Reporting Items for Systematic Reviews and Meta-Analyses (PRISMA) guidelines. A comprehensive literature search was performed across PubMed, Scopus and Embase databases. Studies examining quality management or quality improvement interventions in poisoning care were included.

**Results:**

A total of 5,006 records were identified through database searching. After removal of duplicates, 3057 unique articles remained. During title and abstract screening, 2464 articles were excluded. Full-text assessment led to the exclusion of an additional 578 studies. Ultimately, 15 studies met the inclusion criteria and were included for data extraction and qualitative synthesis.

**Conclusion:**

In the context of increasing clinical and systemic challenges in poisoning management, the implementation of structured quality management strategies is essential. QI interventions may have the potential to enhance patient outcomes, reduce complications, and shorten hospital length of stay, thereby strengthening healthcare system efficiency.

**Trial registration:**

The protocol was registered in the International Prospective Register of Systematic Reviews (PROSPERO) under the registration number CRD42024581815.

**Clinical trial number:**

Not applicable.

**Supplementary Information:**

The online version contains supplementary material available at https://doi.org/10.1186/s12913-026-15038-4.

## Background

The term ‘*poison’* is defined as any chemical substance capable of harming or altering normal human physiology through local or systemic cellular damage resulting from its chemical activity [1]. Poisoning represents a major global public health concern and is among the most frequent causes of emergency department (ED) visits. Its consequences include substantial mortality, morbidity, prolonged hospitalization, and significant economic burden on healthcare systems. Despite its clinical importance, the true incidence of poisoning remains poorly understood due to widespread underreporting and inadequate surveillance mechanisms [2]. Globally, approximately 370,000 deaths have been attributed to intentional poisoning [3]. In addition, 193,469 cases of unintentional poisoning have been documented, with the highest incidence occurring among children under five years of age. In contrast, intentional poisonings are more prevalent among adolescents aged 13 to 19 years [4, 5]. Notably, more than 80% of poisoning-related incidents are reported from low- and middle-income countries (LMICs) [6]. According to the World Health Organization (WHO), over 5% of poisoning-related deaths occur in India, Nepal, and South Korea [7].

The epidemiology of poisoning varies considerably across countries and geographic regions. In nations where agriculture constitutes the primary economic activity, pesticide poisoning is reported as the leading cause of suicide attempts [8]. Globally, more than 20% of poisoning cases are attributed to pesticide exposure, particularly in LMICs, where access to highly hazardous pesticides remains widespread and regulatory enforcement is often inadequate [6]. Effective management of poisoned patients is influenced by multiple clinical, systemic, and socioeconomic factors. These include poor regulatory control, delayed presentation to healthcare facilities, prolonged duration of hospitalization, mode and severity of exposure, treatment-related constraints, delayed diagnosis, and patient-related factors such as age [9]. Compounding these challenges is the limited availability of essential antidotes, often due to high procurement costs and inadequate inventory management in healthcare facilities [10]. In clinical practice, ED physicians frequently depend on reference resources to manage complex or uncommon poisoning cases. However, access to Poison Information Centres (PICs) as well as their consistent availability and provision of up-to-date clinical guidance remains limited, despite physicians possessing substantial experiential knowledge in managing poisoned patients [11]. Consequently, clinical decision-making is further complicated by regional variations in poisoning patterns, infrequent guideline updates, and the scarcity of high-quality evidence-based research supporting antidote use [12]. As a result, treatment recommendations are often derived from case reports or theoretical clinical reasoning rather than robust experimental data [13].

These limitations highlight significant gaps in poisoning care delivery and underscore the urgent need for system-level interventions. Establishing and evaluating quality improvement (QI) programmes in poisoning management is therefore essential. Strengthening toxicovigilance systems through coordinated antidote stewardship, development of standardized treatment protocols, promotion of evidence-based decision-making, and enhancement of healthcare professionals’ competencies can substantially improve patient outcomes. Although such approaches have been adopted in several LMICs, notable deficiencies persist in their implementation, integration, and sustainability. In this context, the purpose of the present systematic review is to identify and synthesize quality improvement strategies implemented across diverse clinical settings to enhance the management of poisoned patients. The review further examines their effectiveness, associated challenges, and facilitating factors to identify priority areas for tailored intervention strategies. Ultimately, the findings aim to inform healthcare professionals, policymakers, programme managers, and planners in strengthening case management practices and implementing effective interventions to reduce the preventable clinical and economic burden of poisoning within healthcare systems.

## Methods

This systematic review is conducted according to the Preferred Reporting Items for Systematic Reviews and Meta-Analyses (PRISMA) 2020 guidelines [14]. The protocol was registered in the International Prospective Register of Systematic Reviews (PROSPERO) under the registration number CRD42024581815 prior data extraction to ensure methodological transparency and rigor [15].

### Search databases and search strategy

The literature focusing on the identification and reporting of quality improvement strategies implemented across healthcare settings was retrieved from databases including PubMed, SCOPUS and Embase. A comprehensive literature search was conducted using the Population, Exposure, Comparator, Outcome (PECO) framework to ensure a structured and systematic approach (Table 1). The search strategy was developed using the keywords *“poisoning*,*” “quality improvement*,*”* and *“management*,*”* combined with appropriate Boolean operators (OR, AND). The complete PubMed search strategy is provided in Supplementary File S1.

**Table 1:** PECO framework used for literature search

Framework	Description
Population	Patients exposed to poisoning, substance abuse, or drug overdose, either accidentally or intentionally
Exposure	Exposure to toxic substances or drugs resulting in acute poisoning or overdose, including intentional and unintentional events
Comparator	Standard care or usual clinical management (where applicable)
Outcome	Implementation and effectiveness of quality improvement strategies in the management of poisoned patients

### Inclusion and exclusion criteria

The identified records were imported into Microsoft Excel 2016, where duplicate records were removed. The remaining records were screened according to the predefined eligibility criteria (Table 2).

Two reviewers (PS and VS) independently screened the records in two stages: (i) title and abstract screening, followed by (ii) full-text assessment for eligibility. Any discrepancies between the reviewers were resolved through discussion or, when necessary, by consultation with a senior researcher (GT and/or RR).

Studies describing QI strategies in any form for the management of poisoning were considered eligible for inclusion. We included studies evaluating QI interventions in poisoning management that reported measurable implementation outcomes, such as clinical process changes, patient outcomes, or system-level performance indicators. Studies describing clinical practice guidelines, consensus statements, or expert recommendations were excluded unless they incorporated an evaluative component assessing implementation impact or clinical effectiveness. No restrictions were applied with respect to publication date or language. Non-English language articles were translated using Microsoft Translator, and the accuracy of translations was verified by a local language expert.

**Table 2:** Inclusion and exclusion criteria for study selection

Inclusion criteria	Exclusion criteria
Patients of any age exposed to poisoning, toxic substances, drug abuse, or drug overdose (accidental or intentional)	Studies focusing on non-poisoning conditions (e.g., trauma, infections, chronic diseases without poisoning component)
Acute poisoning or drug overdose, including pharmaceutical, chemical, household, environmental, or substance-related poisoning	Chronic toxicity without acute management focus; animal or laboratory-only poisoning studies
Outcomes related to improvement in poisoning management (e.g., reduced mortality, improved treatment timeliness, adherence to guidelines, reduced length of stay, improved documentation or reporting)	Studies not reporting outcomes relevant to quality improvement in poisoning management
Any healthcare setting including emergency departments, hospitals, poison control centers, and intensive care units	Community-only studies without healthcare system involvement
Studies published in any language (with translation where required)	Studies with inaccessible full text

### Data extraction

Data were extracted from the included studies using standardized data extraction forms developed in Microsoft Excel 2016. The structure and variables included in the extraction form are provided in Supplementary File S2. The extracted variables included study characteristics (authors, year of publication, country, and study duration), sample size, participant demographics, study design, quality improvement strategies, outcomes assessed, measurement tools, intervention impact, key findings, reported limitations, and study conclusions. In addition, a ‘Level of Evidence’ was assigned to each included study based on study design, using a hierarchy adapted from the Oxford Centre for Evidence-Based Medicine (OCEBM).

Two independent researchers (PS and VS) manually performed the data extraction. During the data reconciliation phase, any disagreements or discrepancies were resolved through discussion or, when necessary, by consultation with a senior researcher (GT and/or RR). The data extractors were familiar with the objectives of the review to ensure accuracy and appropriate contextual interpretation of the findings. To maintain consistency and data quality, clear extraction guidelines were provided, and regular team meetings were conducted to reinforce adherence to standardized procedures based on the predefined eligibility criteria.

### Quality appraisal of the selected studies

The methodological quality of the included studies was assessed according to study design using standardized and validated appraisal tools. Randomized controlled trials (RCTs) were evaluated using the Cochrane Risk of Bias (RoB) tool [16]. Cross-sectional studies were assessed using the Modified Newcastle–Ottawa Scale [17], while cohort and longitudinal studies were appraised using the Risk Of Bias In Non-randomized Studies of Interventions (ROBINS-I, Version 2) tool [18]. Qualitative studies were evaluated using the Critical Appraisal Skills Programme (CASP) checklist [19].

Each tool assessed key domains related to study quality and risk of bias, including participant selection, comparability of study groups, blinding, outcome measurement, completeness of outcome data, and selective reporting. Quality appraisal was conducted independently by two reviewers (PS and VS). The study authors were contacted for any kind of missing information or clarification. Any disagreements were resolved through discussion or, when necessary, through consultation with a third reviewer (GT and/or RR) to achieve consensus.

### Summarising and reporting the results

A meta-analysis was not conducted due to substantial heterogeneity among the included studies with respect to study design, quality improvement strategies, reported outcomes, and clinical contexts. Therefore, a narrative synthesis of the findings was performed. The studies were systematically summarized and grouped according to the type of quality improvement strategy, healthcare setting, and outcome measures. Quality improvement strategies were categorized by intervention type, including education-based interventions, clinical audit mechanisms, guideline development, and multidisciplinary care models.

Quantitative outcomes were summarized descriptively using frequencies, ranges, and direction of effect (improvement, no change, or mixed results). Where available, changes in clinical outcomes such as mortality, length of hospital stay, treatment timeliness, guideline adherence, and documentation quality were reported. Similarly, qualitative findings were synthesized thematically to identify common facilitators, barriers, and implementation challenges associated with quality improvement initiatives in poisoning management. The results were interpreted in the context of study quality and risk-of-bias assessments to ensure balanced conclusions.

## Results

### Study selection

A total of 5,006 records were identified through the literature search. After removal of duplicates, 3,057 unique records remained for screening. During the first stage (title and abstract screening), 2,464 records were excluded. In the second stage (full-text screening), further 578 articles were excluded based on the predefined eligibility criteria. Ultimately, 15 studies were included for data extraction and synthesis [20–34].

**Fig. 1:**
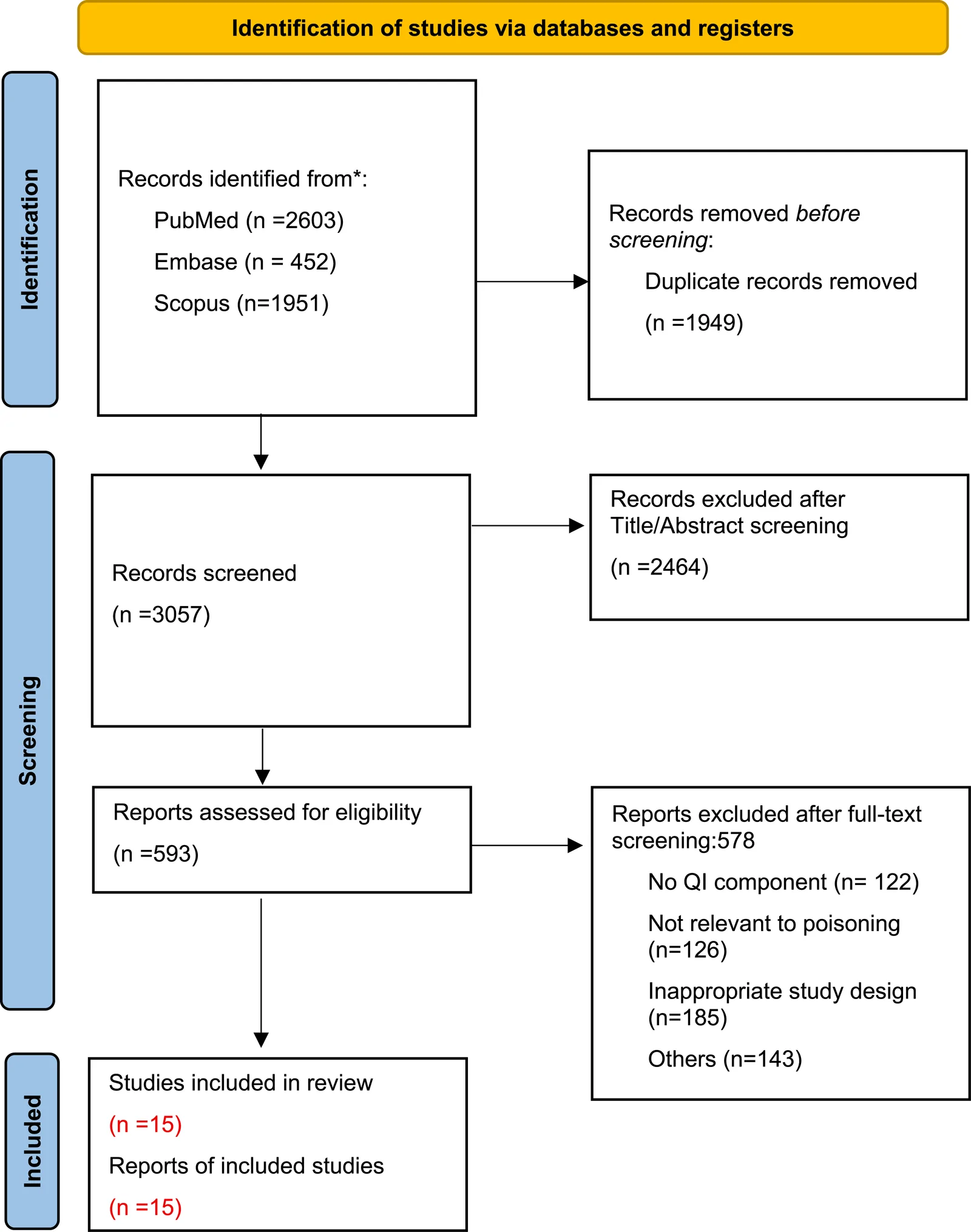
Description of included studies according to PRISMA 2020

Figure 1 presents the PRISMA flow diagram illustrating the process of study identification, screening, eligibility assessment, and inclusion.

### Summary and characteristics of selected studies

The 15 included studies comprised a total of 5,648 participants, with sample sizes ranging from 32 to 1,557. The studies were published between 2006 and 2025. Most studies were conducted in the United States of America (USA) (*n* = 5), followed by multi-site consensus studies conducted in the USA and Canada (*n* = 1), UK (*n* = 1), Spain (*n* = 2), and Australia (*n* = 2). The remaining studies were conducted in Canada, the United Kingdom, Sri Lanka, and Nepal.

Among the included studies, one was a cluster randomized controlled trial, one quasi-experimental study, five were cohort studies, three were cross-sectional studies, three were qualitative studies, and two were longitudinal studies. The study characteristics, including study identification number, study design, study duration, population, sample size, gender distribution, age range, and mean age, are presented in Table 3.

**Table 3:** Characteristics of included studies

Ref	Study ID	Study Design	Age	Gender	Study Duration	Study Population	No. of Participants	Level of Evidence*
[20]	Summerlin JA et al. 2023	Retrospective cohort study	Pre-intervention mean age: 38 ± 20; Post-intervention mean age: 45 ± 22	Male 24 (28%), Female 60 (71.4%)	NR	Patients receiving IV NAC for acetaminophen toxicity	84	II
[21]	Ornato JP et al. 2023	Retrospective cohort study	Mean age: 40.7 years	Male 405 (69.9%), Female 177 (30.4%)	3 years	Patients presenting with poisoning	582	II
[22]	Downes MA et al. 2017	Retrospective cohort study	2009: Median 33 (IQR 23–44). 2012: Median 34 (IQR 23–46)	2009: Male 299 (37.6%), Female 496 (62.4%) 2012: Male 275 (36.1%), Female 487 (63.9%)	NR	Adult patients presenting to ED of tertiary toxicology unit	2009: 795 2012: 762	II
[23]	Martínez-Sánchez L et al. 2020	Observational cross-sectional study	Median age: 14 years (IQR 7–16)	Male 131 (51.6%), Female 123 (48.4%)	12 months (June 2016–May 2017)	Poisoned patients receiving prehospital emergency care	254	IV
[24]	Greene SL et al. 2008	Prospective observational audit with educational intervention (longitudinal study)	NR	NR	12 months	Patients presenting with poisoning	1,101 ED medical and nursing records	IV
[25]	Martínez-Sánchez L et al. 2021	Delphi consensus approach	NR	NR	6 months	Paediatricians	32	V
[26]	Shahmy S et al. 2023	Cluster randomized controlled trial	Detailed age distribution reported for intervention and control groups	Intervention: Male 606 (59.3%), Female 415 (40.6%); Control: Male 683 (58.6%), Female 482 (41.3%)	One year following one-day educational workshop	Hospital-based poisoning management	Pre-test: 50; Post-test: 34	I
[27]	Thomas A et al. 2023	Simulation-based education (cross-sectional study)	NR	NR	3 months (8 simulation sessions)	Medical residents	53 residents (18 PGY-1, 17 PGY-2, 18 PGY-3)	IV
[28]	St-Onge M et al. 2012	Retrospective cohort study	Mean age: females 56 years; males NR	Male 42 (41%), Female 61 (59%)	34 months	Calcium channel blocker poisoning patients	103	II
[29]	Chyka PA et al. 2006	Retrospective observational (longitudinal cohort study)	NR	NR	12 months pre- and post-intervention	Poison control center exposure reports	17,179 (pre); 17,498 (post)	II
[30]	Woolf AD et al. 2007	Retrospective and prospective audit (longitudinal study)	NR	NR	NR	NR	Not reported	IV
[31]	Bhattarai MD et al. 2010	Retrospective and Prospective audit (longitudinal study)	NR	NR	Pre-intervention: 2 months; Post-intervention: 2 months	Postgraduate residents	Pre: 168; Post: 126	IV
[32]	Hurley SP et al. 2025	Retrospective cross-sectional study	Median age: 37 years (IQR 23–50.3)	Male 16 (21.4%), Female 59 (78.6%)	3 months	Patients	75	IV
[33]	Dart RC et al. 2023	Delphi consensus approach	NR	NR	2 years	Expert panelists	21	V
[34]	Pettie J et al. 2012	Quasi experimental study	pre-ICP study group, mean: 33 (range, 14–94) post-ICP group mean: 33 (range, 14–72)	pre-ICP study group: Male 47 (29%), Female 114 (71%) post-ICP group: Male 34 (30%) Female 79 (70%)	18 months	Patients with paracetamol poisoning	274	III

NR- Not Reported, IV-Intravenous, NAC- N-Acetylcysteine, IQR- Interquartile range, ED- Emergency department, PGY- Postgraduate year, ICP- Integrated care pathway

* Level of Evidence assigned based on a hierarchy adapted from the OCEBM, according to study design (Level I = highest, Level V = lowest)

### Methodological quality assessment of the included studies

Of the 15 included studies, one was a cluster RCT; therefore, this study was evaluated using the Cochrane RoB assessment tool [26]. Overall, the study demonstrated a low risk of bias across most domains, including outcome measurement, selective reporting, and missing outcome data. Some concerns were identified in the randomization domain. Since the intervention was educational in nature, blinding of healthcare professionals is not feasible and is unlikely to significantly affect the study outcomes. One study, which was quasi-experimental in design [34], demonstrated a serious risk of bias, particularly due to issues in selection of participants and confounding factors. These limitations are inherent to the non-randomized, before–after nature of the study. Consequently, the overall risk of bias was judged to be serious.

Among the seven cohort and longitudinal studies assessed using the ROBINS-I tool, five studies demonstrated a serious overall risk of bias [20–22, 24, 31], while two studies were assessed as having a critical risk of bias [28, 29]. None of the studies were judged to have a low or moderate risk of bias.

The three included cross-sectional studies were assessed using the Modified Newcastle–Ottawa Scale. Each study was evaluated according to the predefined criteria and categorized as *very good* (9 points), *good* (7–8 points), *satisfactory* (5–6 points), or *unsatisfactory* (0–4 points). Overall, two studies [23, 32] received a *good* quality rating with scores of 8 and 7 points, respectively, while one study [27] was rated *unsatisfactory* with a score of 3 points. None of the cross-sectional studies were rated as very good or satisfactory.

The three qualitative studies were appraised using the CASP checklist. Each study was evaluated for methodological rigor, validity, and trustworthiness of findings by summarizing key elements under the categories of *methodologically sound (positive)*, *poor or negative methodology*, and *unclear methodology*. In accordance with CASP guidance, numerical scoring was not applied, instead narratively summarised. All three qualitative studies [25, 30, 33] clearly defined their research aims, employed appropriate expert panel selection, and utilized a structured consensus process based on the Delphi methodology. The findings were clearly reported and demonstrated strong clinical relevance. One study highlighted existing knowledge gaps and proposed directions for future research, while another emphasized the importance of avoiding outdated or potentially harmful clinical interventions. None of the three studies [25, 30, 33] explicitly addressed researcher reflexivity, potential conflicts of interest, or methods used to minimize bias. Additionally, two studies contributed to potential selection bias by failing to report clear criteria for literature inclusion. In one study [26], the consensus process focused primarily on Spanish clinical practice, making it unclear whether diverse international perspectives were adequately represented (Table 4).

**Table 4:** Overall risk of bias assessment of the studies

Study ID	Study Design	Risk of bias tool used	Risk of bias
Shahmy S et al. 2023 [26]	Cluster randomized controlled trial	Rob 1	Low risk
Greene SL et al. 2008 [24]	Cohort	ROBINS I V2	Serious
Downes et al. 2017 [22]	Cohort	ROBINS I V2	Serious
Bhattarai MD et al. 2010 [31]	Cohort	ROBINS I V2	Serious
Chyka PA et al. 2006 [29]	Cohort	ROBINS I V2	Critical
Summerlin JA et al. 2023 [20]	Cohort	ROBINS I V2	Serious
Ornato, JP et al. 2022 [21]	Cohort	ROBINS I V2	Serious
St-Onge M et al. 2012 [28]	Cohort	ROBINS I V2	Critical
Martínez-Sánchez L et al. 2020 [23]	Cross-sectional	Modified New Castle Ottawa	Good
Hurley SP et al. 2025 [32]	Cross-sectional	Modified New Castle Ottawa	Good
Thomas A et al. 2023 [27]	Cross-sectional	Modified New Castle Ottawa	Unsatisfactory
Woolf AD et al. 2007 [30]	Qualitative	CASP Checklist	Low risk
Dart RC et al. 2023 [33]	Qualitative	CASP Checklist	Low risk
Martínez Sánchez L et al. 2021 [25]	Qualitative	CASP Checklist	Low risk
Pettie J et al. 2012	Quasi-experimental	ROBINS I V2	Serious

### Quality improvement strategies in poisoning management

Included 15 studies reported a wide range of QI strategies across various healthcare settings for the management of poisoning cases across emergency departments, inpatient units, poison control centres and other hospital services. The QI strategies were classified into five major domains such as (i) education and training-based (ii) audit and feedback mechanisms (iii) guideline or consensus development (iv) clinical pathway or protocol optimisation and (v) system-level process measures. Certain studies have more than one intervention component, reflecting multifaceted quality improvement initiatives for the management of poisoning. Despite the presence of overlap, the studies are categorised based on the primary intervention for the ease of data synthesis (Table 5).

**Table 5:** A matrix map of intervention strategies mentioned across the studies

Study ID	Education/ Training	Audit and feedback	Guideline/ consensus development	Clinical pathway/ protocol optimisation	System-level process measures
Summerlin JA et al. 2023 [20]	✓	-	-	✓*	-
Ornato JP et al. 2023 [21]	-	-	-	✓*	✓
Downes MA et al. 2017 [22]		✓		✓*	✓
Martínez-Sánchez L et al. 2020 [23]	-	-	-	-	✓
Greene SL et al. 2008 [24]	✓*	✓*	-	-	✓
Martínez-Sánchez L et al. 2021 [25]	-	-	✓*	-	✓
Shahmy S et al. 2023 [26]	✓*	-	✓	-	-
Thomas A et al. 2023 [27]	✓*	-	-	-	-
St-Onge M et al. 2012 [28]	-	✓	-	-	✓*
Chyka PA et al. 2006 [29]	-	-	-	-	✓*
Woolf AD et al. 2007 [30]	-	-	✓*	-	-
Bhattarai MD et al. 2010 [31]	-	✓*	-	-	✓
Hurley SP et al. 2025 [32]	-	✓*	-	-	✓
Dart RC et al. 2023 [33]	-	-	✓*	-	-
Pettie J et al. 2012 [34]	✓	-	✓	✓*	-

✓ = component present, – = component not present. *Primary intervention

### Education/training-based interventions

In the present systematic review, education and/or training-based interventions refer to structured educational activities aimed at improving healthcare quality through enhancement of staff knowledge, utilising the clinical skills in care delivery, and promotion of behavioural capacity building as part of professional development as well as a quality improvement strategy in the management of poisoning. Three studies utilised education and training as a QI strategy to enhance the management of poisoned patients, including one longitudinal study [24], one cluster RCT [26], and one cross-sectional study [27]. The longitudinal study by Greene SL et al. focused on the development of an electronic clinical toxicology database, followed by the systematic analysis of patient records to identify and evaluate pitfalls in poisoning management. The identified shortcomings were subsequently addressed through bimonthly audits and structured educational programmes designed and delivered by emergency and toxicology service staff within a tertiary care setting. The strategy resulted in a reduced incidence of predefined management pitfalls. In addition, the targeted educational interventions delivered using real-life, case-based scenarios were effective in identifying knowledge gaps and improving clinicians’ ability to manage poisoned patients. Overall, the study demonstrated that the establishment of a dedicated toxicology database for systematic case collection, combined with a continuous education programme for emergency service staff, significantly reduced shortfalls and improved the quality of hospital management for acutely poisoned patients [24].

A cluster RCT conducted in rural Sri Lankan hospitals by Shahmy et al. evaluated the effectiveness of an educational intervention in improving compliance with national guidelines for snakebite management. A total of 44 hospitals were randomised into an intervention group (*n* = 24), which received a brief educational programme based on the Sri Lankan snakebite management guidelines, and a control group, in which educational promotion was restricted. The guidelines themselves were accessible to both groups. Although quality of care indicators, clinical documentation, and appropriate referral to higher-level hospitals remained statistically insignificant between the two groups, the intervention group demonstrated a significant increase in knowledge following completion of the one-day workshop (*p* < 0.0001). Given the high baseline rate of satisfactory responses in both groups, measurable clinical improvement was not observed. The authors concluded that further research was required to determine whether educational interventions alone can translate knowledge gains into improved clinical outcomes [26].

Another study by Thomas A et al. demonstrated the effectiveness of simulation-based education within emergency care settings. Simulation-based training was delivered to medical residents focusing on lithium toxicity over a series of eight simulated sessions. The study found that toxicological overdoses were managed more effectively following simulation-based education, indicating an improvement in the quality of care. This educational strategy provided a safe and controlled learning environment that facilitated repetitive practice, enhanced clinical decision-making, and strengthened residents’ confidence in poisoning management. The findings support simulation-based education as an effective QI intervention for improving the management of poisoned patients [27].

### Audit and feedback mechanism

Medical audit refers to a structured seven-step process that focuses on improving patient care through the application of existing evidence, feedback derived from educational activities, and continuous assessment of clinical practice. Audits function both as an intervention and as a method to measure the effectiveness of existing programmes. In this systematic review, three studies utilised prospective audit and feedback as the primary QI intervention to evaluate current practices and indirectly improve system-level process measures [24, 31, 32]. The study by Greene SL et al. employed bimonthly audits as a QI strategy to assess the effectiveness of a clinical toxicology database alongside a continuous medical education programme [24]. Another study by Bhattarai et al. explored a novel “10 PM audit” strategy focusing on residents’ late-evening routine documentation for organophosphate (OP) poisoning. Given that OP poisoning requires meticulous monitoring, inadvertent cessation of atropine during night shifts may lead to the re-emergence of life-threatening symptoms, making effective communication critical in emergency settings. The 10 PM audit significantly improved resident communication from 15.5% to 81.7% (*p* < 0.01) and proved to be an effective quality-of-care strategy in a general practice setting in Nepal [31].

Medical audit is also a valuable documentation tool for assessing poisoning characteristics and adherence to treatment protocols. With a similar objective, Hurley et al. conducted a four-month retrospective audit to evaluate the epidemiological characteristics of paracetamol poisoning within a local health network in South Australia. Management strategies were assessed for compliance with national guidelines. The findings indicate overall adherence to guideline-recommended care; however, deficiencies in clinical documentation and instances of antidote underdosing were identified, highlighting the need for more structured and targeted QI strategies [32].

### Guideline or consensus development

The study by Martínez-Sánchez L et al. described a consensus process undertaken by the Poisoning Working Group of the Spanish Society of Paediatric Emergencies (GTI-SEUP) to develop a “Not-to-do” list for the management of potential toxin exposure. Consensus was achieved using a modified Delphi methodology incorporating a 9-point Likert scale.

The process involved a three-step verification framework, with final agreement reached following structured discussion and systematic assessment of each item. This consensus-driven approach within a dedicated expert working group represented an important quality improvement strategy, particularly in areas where certain interventions may be ineffective, unnecessary, or potentially harmful to the paediatric population. By identifying practices to avoid, the initiative contributed to improving quality of care and reducing low-value or inappropriate interventions [25].

The guideline by Woolf AD et al. outlined a consensus-building process employed by an expert panel assembled by the American Association of Poison Control Centres (AAPCC) to create an evidence-based guideline for the prehospital triage and care of suspected tricyclic antidepressant (TCA) poisoning cases. Consensus was formulated through a consensus-building process that employed an expert-driven approach, including systematic abstraction of relevant literature, iterative guideline development, panel discussion, and incorporation of feedback from secondary reviewers. The process included the identification of key decision points, such as referral criteria, decontamination practices, and other interventions such as sodium bicarbonate or benzodiazepines, with recommendations developed according to the strength of the evidence and expert consensus. This consensus-building process among a multidisciplinary expert panel was an important quality-improvement activity, especially in regions where TCA poisoning often results in rapid disease progression, unnecessary healthcare utilization, or suboptimal prehospital care. This initiative helped improve patient outcomes, reduce unnecessary emergency department visits, and reduce risks associated with TCA toxicity [30].

The consensus statement by Dart RC et al. reported on a consensus-building process conducted by an expert panel assembled by four societies of clinical toxicology in North America, namely America’s Poison Centres, American Academy of Clinical Toxicology (AACT), American College of Medical Toxicology (ACMT), and Canadian Association of Poison Control Centres, to establish harmonized guidelines for acetaminophen poisoning in the US and Canada. Consensus was developed through a Delphi process involving rounds of voting on statements developed from poison centre guidelines and literature review. The process included a non-voting chairperson and 11 rounds of consensus development, requiring at least 75% approval without strong dissent, and also incorporating feedback from external organizations. This consensus-building effort within a multidisciplinary group of experts was an important quality improvement activity, especially in regions where there was potential for inconsistencies in treatment due to practice variability, such as in high-risk cases of poisoning, extended-release formulations, and co-ingestions. Through standardization of patient assessment, decontamination, acetylcysteine treatment, and special considerations for pregnancy or paediatric exposures, this effort helped improve evidence-based practice, minimize disparities in treatment, and enhance overall patient care in acetaminophen poisoning [33].

### Clinical pathway or protocol optimisation

Clinical pathway or protocol optimisation refers to the introduction or modification of interventions within existing clinical frameworks with the aim of enhancing the quality of care in poisoning management. This represents an important QI strategy in emergency and toxicology services.

Summerlin et al. conducted a retrospective pre–post cohort study to evaluate the effectiveness of a pharmacist-led toxicology consultation service for patients receiving IV NAC for acetaminophen toxicity. The primary endpoint was the reduction of prescribing errors and inappropriate therapy. The post-intervention group demonstrated significantly fewer errors compared with the pre-intervention group (*p* = 0.003), supporting the integration of clinical pharmacist–based toxicology consultation teams within emergency services for poisoning management [20].

Another study by Ornato JP et al. introduced a “time-to-treat” analysis using retrospective data on 9-1-1 call receipt and the administration of the first dose of naloxone in adult opioid poisoning cases. Comparisons were made between pre– and post–COVID-19 periods to evaluate the importance of timely antidote delivery and hospital presentation beyond conventional treatment protocol considerations. Although no statistically significant difference was observed between the two periods, the study highlighted the critical role of time-based metrics in emergency poisoning treatment protocols [21].

Downes MA et al. conducted a cohort study assessing the establishment of an Emergency Short Stay Unit (ESSU) for adults poisoned with calcium channel blockers (CCB). The study compared two patient cohorts across different time periods, with hospital length of stay as the primary endpoint. Results demonstrated a significant reduction in length of stay following implementation of the ESSU (absolute difference: 57%; 95% CI: 53–62%; *p* < 0.0001). This QI strategy effectively reduced overall hospitalisation time, facilitated early recovery, and helped mitigate ED overcrowding [22].

A study by Pettie J et al. evaluated the impact of introducing an integrated care pathway (ICP) on the management of patients with paracetamol poisoning [34]. This was a retrospective before–after study, with data collected over two 3-month periods before and after ICP implementation in the emergency department and toxicology unit. The ICP was used in 77% of cases and was associated with significant improvements in care processes, particularly in documentation of patient assessment, which increased from 54% (87/161) pre-ICP to 89% (101/113) post-ICP (*p* < 0.0001). The ICP provided a structured, guideline-based clinical pathway covering assessment, treatment decisions, and NAC administration. After implementation, there were significant improvements in documentation, appropriate blood sampling, correct NAC prescribing, and timely treatment initiation. The study demonstrated that standardized clinical pathways improve adherence to guidelines and overall quality of care in complex poisoning management.

### System-level process measures

In this systematic review, system-level process measures were considered as outcomes rather than interventions when evaluating QI strategies. Several studies either directly assessed system-level process measures or indirectly contributed to process-level changes (Table 5). An example of the former is the study by Chyka et al. in which revised guidelines for the use of ipecac syrup, gastric lavage, cathartics, and activated charcoal had already been implemented by the poison control centre within the healthcare system. These guidelines were developed as part of an earlier QI initiative, and their effectiveness was subsequently evaluated using a structured quality improvement framework. Given the longstanding debate regarding the clinical value and appropriateness of gastric decontamination methods, this study sought to introduce a process-level shift aimed at improving the quality of care in poisoning management. The primary objective was to assess post-implementation adherence to the updated guidelines rather than to alter or challenge individual physician decision-making. The revised recommendations guided treatment selection without adversely affecting patient outcomes, thereby demonstrating how system-level process optimisation can support evidence-based practice in poisoning care [29].

Similarly, the study by St-Onge et al. evaluated physician compliance with recommendations provided by the Poison Control Centre (PCC) for CCB poisoning. Retrospective analysis demonstrated that only 42% of the 103 prescriptions were compliant with PCC recommendations, and among these, 81% involved formal consultation with the PCC. The study identified several barriers to protocol adherence that contributed to inappropriate prescribing, polypharmacy, increased hospital length of stay, and prolonged use of intravenous therapies. These findings highlight that the identification of systemic barriers and the implementation of system-level transitions are as critical as the introduction of new interventions for improving the quality of care in poisoning management [28].

In summary, the studies included in this review employed a wide range of quality improvement strategies for poisoning management, including guideline development, educational interventions, compliance assessment, audit and feedback mechanisms, pharmacist-led consultations, and organisational restructuring. While multicomponent interventions demonstrated improvements in clinician knowledge, guideline adherence, and patient outcomes, others revealed persistent gaps in protocol compliance and prehospital care. Key barriers and facilitators for the implementation of QI strategies are summarised in Table 6. However substantial heterogeneity present in the studies limit for the quantitative analysis across the studies. Furthermore, while several studies reported improvements in clinical or process outcomes, it is important to note that the majority were assessed as having ‘Serious’ or ‘Critical’ risk of bias which limits the certainty of these findings. Collectively, these findings underscore the ongoing need for targeted, system-focused quality improvement initiatives to optimise the management of poisoned patients.

**Table 6:** Barriers and facilitators identified in the management of poisoning

Sl. No	Barriers	Facilitators	Studies supporting
1.	Limited Training and awareness, High staff turnover	Education and Training	[24, 26, 27]
2.	Time constraints in emergency care	Integration into workflow, Multidisciplinary collaboration	[20–22]
3.	Poor documentation, Lack of monitoring, and follow-up	Audit and feedback	[24,26,31,32]
4.	Resistance to practice change	Leadership and institutional support	[24,29]
5.	Limited generalisability	Standardised clinical guidelines	[25,30,33,34]

## Discussion

The purpose of this systematic review was to evaluate the QI strategies employed to enhance the care of patients presenting with poisoning. The findings demonstrate that QI strategies play a critical role in improving toxicological management, optimizing patient outcomes, and promoting the rational use of therapeutic resources and antidotes. The included studies assessed a broad range of interventions, including the development of expert consensus recommendations [25, 30, 33], educational programs [24, 26, 27], compliance evaluations [28, 32], revised clinical guidelines [29, 34], medical audits [31], pharmacist-led toxicology consultation services [20], time-to-treatment analyses [21], and the establishment of specialized emergency toxicology units [22]. However, the strength of the evidence is limited by the methodological quality across the included studies. Despite methodological heterogeneity, most studies consistently reported improvements in clinical processes or patient outcomes, underscoring the growing importance of QI initiatives in toxicological stewardship.

Our findings align with previous literature emphasizing the value of structured, evidence-based approaches in poisoning management. Ghannoum and Roberts (2023) highlighted that a systematic framework incorporating early intervention, antidote stewardship, and informed clinical decision-making significantly improves outcomes in toxicological emergencies [35]. Similarly, Blanchard et al. (2019) identified considerable variation in physician experience and treatment decisions during poisoning management in India, underscoring the need for standardized training and institutional protocols [36]. Comparable deficiencies were reported by Liu et al. (2016), who demonstrated wide disparities among Chinese emergency physicians regarding antidote knowledge and availability, reflecting global challenges in toxicology education and antidote accessibility [37].

Pharmacist-led toxicology consultation services have emerged as an effective QI intervention. Summerlin et al. demonstrated that pharmacist involvement significantly improved the appropriateness of N-acetylcysteine use in acetaminophen toxicity, highlighting the expanding role of pharmacists in toxicological stewardship [20]. Nevertheless, economic constraints remain substantial. Heindel et al. reported escalating antidote costs in the United States, while García-Martín and Torres Santos-Olmos [38] emphasized the importance of maintaining minimum antidote stocks and establishing clear usage guidelines to ensure both clinical readiness and cost-effectiveness. These interventions align with the concept of toxicological stewardship, which emphasizes the rational, evidence-based use of antidotes and poisoning management resources. Pharmacist involvement has been shown to improve adherence to treatment protocols, reduce medication errors, and enhance decision-making in complex poisoning scenarios [39]. An important advantage of such interventions is their potential cost-effectiveness, particularly in the context of high-cost antidotes such as fomepizole and digoxin-specific antibody fragments. Inappropriate initiation, excessive dosing, or delayed discontinuation of these therapies can contribute significantly to healthcare expenditure. Pharmacist-led review and protocol-driven care pathways can mitigate these issues by ensuring appropriate patient selection, accurate dosing, and timely reassessment of ongoing need.

The implementation of organized toxicological stewardship programs offers significant potential for standardizing poisoning care and improving patient outcomes. Core components include essential antidote stocking, structured staff education, pharmacist-led consultation services, regular audits with feedback, evidence-based clinical guidelines, and specialized short-stay or observation units. Integration of electronic health records with toxicovigilance databases may further enhance hospital preparedness and enable continuous monitoring, data-driven evaluation, and sustained quality improvement in acute toxicological emergencies [30, 32, 35]. Furthermore, structured toxicology stewardship programs can help prevent “antidote wastage” by reducing unnecessary administration, avoiding duplication of therapy, and promoting adherence to evidence-based thresholds for treatment initiation. This is particularly relevant in resource-constrained settings, where optimizing the use of limited and costly antidotes is critical [40]. Incorporating toxicological stewardship into routine clinical practice may therefore represent a sustainable and scalable strategy for improving poisoning management across diverse healthcare settings. In an era of rising antidote costs, integrating toxicological stewardship into clinical pathways offers a pragmatic approach to balancing clinical efficacy with economic sustainability.

Structural and organizational interventions have also demonstrated measurable benefits. Martínez-Sánchez et al. reported improvements in prehospital and emergency care pathways for paediatric poisonings [23], while Downes et al. [22] showed that Emergency Short-Stay Units enhanced both efficiency and safety in the management of poisoned children. Despite these advances, adherence to established clinical guidelines remains suboptimal. St-Onge et al. [28] and Hurley et al. [32] documented inadequate compliance with recommended management protocols for calcium channel blocker and paracetamol poisoning, respectively. In contrast, targeted educational initiatives and audit-based feedback interventions, such as those described by Greene et al. [24] and Shahmy et al. [26], were associated with substantial improvements in clinical adherence and patient outcomes.

In addition to structural quality improvement interventions, the rapid digital transformation of healthcare systems is increasingly influencing the delivery of poisoning management services. Digital health tools such as electronic health records, clinical decision support systems, telemedicine platforms, and toxicology databases can enhance the timeliness, accuracy, and consistency of care in acute toxicological emergencies. These systems facilitate rapid access to antidote dosing guidelines, support real-time toxicovigilance, and improve coordination between emergency physicians, intensivists, and clinical pharmacists. Furthermore, integration of digital platforms can strengthen antidote inventory management and reduce delays in time-sensitive interventions, thereby improving patient outcomes [41, 42]. However, despite these advantages, inequities in digital infrastructure and variability in digital literacy among healthcare providers may limit uniform adoption. From a knowledge management perspective, fragmented data systems and poor interoperability may further restrict the effective translation of digital information into clinical decision-making. Therefore, strengthening digital literacy, ensuring robust infrastructure, and developing integrated, user-friendly toxicology information systems are essential to fully realize the potential of digital transformation in poisoning care [42].

Alongside digital and system-level influences, structured clinical governance approaches such as consensus-based recommendations and stewardship frameworks play a critical role in standardizing toxicology care. Consensus-based and “not-to-do” recommendations have increasingly evolved into proactive stewardship tools [23, 30]. Dart et al. [33] further demonstrated the benefits of harmonizing international guidelines for acetaminophen toxicity management. However, the implementation of such frameworks remains limited in low- and middle-income countries. Blanchard et al. [36] and Kumar et al. [11] highlighted systemic deficiencies in tertiary healthcare institutions and advocated for the establishment of dedicated Poison Information Centres to strengthen clinical management, professional education, and national toxicovigilance systems.

Despite increasing recognition of toxicology-focused QI initiatives, substantial evidence gaps remain, particularly in LMICs where poisoning incidence is high. Only a limited number of studies represented LMIC settings [26, 31], highlighting a significant gap in context-specific evidence. A clear distinction was observed between interventions conducted in LMICs and those implemented in high-income settings. The cluster randomized trial conducted in rural Sri Lankan hospitals demonstrated a significant improvement in healthcare providers’ knowledge following an educational intervention; however, this did not translate into measurable clinical improvements [26]. In contrast, studies conducted in high-income settings, such as those evaluating structured clinical pathways and pharmacist-led toxicology consultation services in the United States, reported measurable improvements in clinical outcomes, including reduced prescribing errors and improved treatment efficiency. Several factors may explain this discrepancy. In LMIC settings, systemic constraints such as limited availability of antidotes, inadequate diagnostic infrastructure, high patient load, and variability in healthcare workforce training may hinder translation of knowledge into practice. Additionally, organizational and system-level barriers, including lack of standardized protocols, limited access to toxicology support services, and delayed patient presentation, may further reduce effectiveness. Conversely, high-income settings benefit from well-established infrastructure, integrated clinical pathways, and multidisciplinary support systems, facilitating successful implementation and sustainability of QI strategies [43]. These findings suggest that educational interventions alone may be insufficient in resource-limited settings and should be complemented by system-level strengthening to achieve meaningful clinical impact.

The findings were also interpreted based on the relative strength of the QI interventions, as described in established frameworks such as the action hierarchy. Besides the level of evidence, interventions could be broadly categorized into lower-strength strategies that rely on individual behavior change (e.g., education and training) and higher-strength strategies that involve system-level modifications such as standardization, clinical pathways, decision support tools, and workflow redesign. In the present review, a substantial proportion of included studies focused on education- and training-based interventions. While these approaches improved knowledge and awareness, their impact on clinical outcomes was variable and often limited in sustainability. In contrast, higher-strength interventions such as integrated care pathways, pharmacist-led toxicology consultation services, and organizational restructuring (e.g., emergency short-stay units) demonstrated more consistent and sustained improvements in clinical and process outcomes. These findings suggest that interventions embedded within clinical systems and workflows are more likely to produce durable improvements compared to those relying primarily on individual-level changes. Furthermore, given that many included studies were multifaceted, the observed effectiveness may reflect the combined influence of both lower- and higher-strength components, rather than any single intervention alone.

In addition to these research gaps, several methodological limitations of the present review should be acknowledged. The literature search was restricted to three electronic databases, which may have resulted in the omission of relevant studies. However, the use of major biomedical databases and a comprehensive search strategy incorporating MeSH terms strengthens methodological rigor. Inclusion of multiple languages reduced language bias, although exclusion of grey literature and conference abstracts may have limited evidence breadth. A key limitation of this review is the overall low quality of the included evidence. Of the seven cohort studies, five were assessed as having ‘Serious’ risk of bias and two as ‘Critical’ risk using ROBINS-I. Within an evidence-based medicine framework, this limits the confidence in observed effects and precludes strong causal inferences. As such, findings should be interpreted with caution and highlight the need for well-designed prospective studies to evaluate QI interventions in poisoning management. Given these contextual disparities and evidence limitations, future research should focus on evaluating the long-term sustainability, cost-effectiveness, and patient-centered outcomes of stewardship interventions. Multicentre, mixed-methods studies incorporating the perspectives of clinicians, pharmacists, and health administrators may provide valuable insights into context-specific implementation strategies.

### Implications for practice and policy

The findings of this systematic review highlight important considerations for translating QI strategies into clinical practice and health system planning. While a wide range of interventions have been implemented across different settings, their effectiveness varies substantially depending on their position within the QI intervention hierarchy.

Education- and training-based interventions were the most frequently reported strategies. Although these approaches improved knowledge and awareness among healthcare professionals, their impact on clinical outcomes was inconsistent when implemented in isolation. This reflects their reliance on individual behavior change, which may limit sustainability, particularly in high-pressure environments such as emergency care. In contrast, higher-strength interventions such as standardized clinical pathways, protocol optimization, pharmacist-led toxicology consultation services, and system-level process redesign demonstrated more consistent and sustained improvements in both clinical and process outcomes. These strategies reduce reliance on individual decision-making by embedding evidence-based practices into routine workflows, thereby enhancing reliability and reducing variability in care. Multifaceted interventions that combined education with system-level components (e.g., audit and feedback, clinical protocols, and multidisciplinary collaboration) were particularly effective. These approaches addressed both knowledge gaps and structural barriers, suggesting that integrated strategies are essential for achieving meaningful and sustainable improvements in poisoning management.

From a practical standpoint, prioritization of interventions should be guided by available resources and healthcare context. High-resource settings should prioritize system-level redesign, including clinical decision support systems, integrated care pathways, and dedicated toxicology services. While resource-limited or rural settings may initially benefit from simplified standardized protocols, targeted education programs, and audit-and-feedback mechanisms, with gradual integration of system-level strategies as capacity improves. Overall, the findings support a shift from predominantly education-based approaches toward more robust, system-oriented QI strategies. Embedding these interventions within routine clinical workflows and aligning them with toxicological stewardship principles may enhance both effectiveness and sustainability. Future efforts should focus on developing scalable, context-specific implementation frameworks and evaluating long-term outcomes, cost-effectiveness, and patient-centered impacts of these interventions.

## Conclusion

This systematic review demonstrates that quality improvement strategies play a pivotal role in strengthening poisoning management and advancing patient safety. The reviewed interventions provide valuable insight into current standards of toxicological care and establish a framework for informed clinical decision-making and improved patient outcomes. However, persistent variability in practice, limited guideline adherence, and disparities in resource availability underscore the urgent need for robust, evidence-based toxicological stewardship programs. Addressing these gaps is essential to optimize clinical care, reduce complications and hospital length of stay, and enhance the overall efficiency and sustainability of healthcare systems managing poisoning incidents. Future research should focus on evaluating the long-term effectiveness, scalability, and cost-efficiency of these interventions, particularly within low- and middle-income settings where the burden of poisoning remains substantial. Higher-quality studies are required before definitive conclusions can be drawn or widespread implementation recommended.

## Supplementary Information

Below is the link to the electronic supplementary material.

Supplementary Material 1

Supplementary Material 2

## Data Availability

The data will be available from the authors upon request.
