# Supplementary material for: Quality improvement strategies in the management of poisoning: a systematic review
Source: BMC Health Serv Res. 2026 Jul 18;26:1410. doi: 10.1186/s12913-026-15038-4 (PMC13673796; doi:10.1186/s12913-026-15038-4)
Supplement: Supplementary file 1 [file 12913_2026_15038_MOESM1_ESM.docx]

**SUPPLEMENTARY FILE**

Supplementary File 1- Search Strategy

A) PubMed

| **Search No.** | **Search Strategy** | **Items Found** |
| --- | --- | --- |
| #1 | ((Patient) OR (Clients)) OR (Client) | 93,65,621 |
| #2 | ((Improvement, Quality) OR (Improvements, Quality)) OR (Quality Improvements) | 2,75,468 |
| #3 | Poisonings | 3,96,130 |
| #4 | poisonous effects | 2,54,148 |
| #5 | (margin of safety) OR (toxic potential) | 9,46,370 |
| #6 | ((Drug Overdoses) OR (Overdose, Drug)) OR (Overdoses, Drug) | 28,095 |
| #7 | (((#3) OR (#4)) OR (#5)) OR (#6) | 12,83,878 |
| #18 | ((#1) AND (#2)) AND (#7) | **2,603** |

B) Embase

| **Search No.** | **Search Strategy** | **Items Found** |
| --- | --- | --- |
| #1 | ‘patient’/exp OR ‘patient’ | 80,54,566 |
| #2 | ‘intoxication’/exp OR ‘intoxication’ | 5,33,606 |
| #3 | ‘total quality management’/exp OR ‘total quality management’ | 1,05,100 |
| #4 | #1 AND #2 AND #3 | **452** |

C) Scopus

| **Search No.** | **Search Strategy** | **Items Found** |
| --- | --- | --- |
| #1 | ( ALL ( "patient" ) OR ALL ( "client" ) OR ALL ( "clients" ) ) | 18,487,199 |
| #2 | ( ALL ( "improvement, quality" ) OR ALL ( "improvements, quality" ) OR ALL ( "quality improvements" ) ) | 395,348 |
| #3 | ALL ("poisonings" ) | 497,619 |
| #4 | ALL ("poisonous effects" ) | 581 |
| #5 | ( ALL ( "margin of safety" ) OR ALL ( "toxic potential" ) ) | 25,777 |
| #6 | ( ALL( "drug overdoses" ) OR ALL ( "overdose, drug" ) OR ALL ( "overdoses, drug" ) ) | 50,230 |
| #7 | #3 OR #4 OR #5 OR #6 | 556,213 |
| #8 | #1 AND #2 AND #7 | **1950** |
